# Effect of different placement techniques on marginal microleakage of deep class-II cavities restored with two composite resin formulations

**DOI:** 10.4103/0972-0707.62633

**Published:** 2010

**Authors:** Radhika M, Girija S Sajjan, Kumaraswamy B N, Neetu Mittal

**Affiliations:** Department of Conservative Dentistry and Endodontics, Sri Sai College of Dental Surgery, Vikarabad, India; 1 Department of Vishnu Dental College, Bhimavaram, Andhra Pradesh, India; 2 Department of Bapuji Dental College and Hospital, Davangere, Karnataka, India; 3 Department of Surendra Dental College and Hospital, Sri Ganganagar, Rajasthan, India

**Keywords:** Class II restorations, flowable liner, marginal microleakage, precured composite insert

## Abstract

**Aim:**

The study aims to evaluate and compare marginal microleakage in deep class II cavities restored with various techniques using different composites.

**Materials and Methods:**

Sixty freshly extracted teeth were divided into six groups of 10 teeth each. Standardized class II cavities were made and were restored using composites of different consistencies with different placement techniques. Group 1 with Microhybrid composite, Group 2 with Packable composite, Group 3 Microhybrid composite with a flowable composite liner, Group 4 Packable composite with a flowable composite liner, Group 5 Microhybrid composite with precured composite insert in second increment and Group 6 Packable composite with precured insert in second increment. Specimens then were stored in distilled water, thermocycled and immersed in 50% silver nitrate solution. These specimens were sectioned and evaluated for microleakage at the occlusal and cervical walls separately using stereomicroscope.

**Results:**

The results demonstrated that in the occlusal wall, packable composite, showed significantly more marginal microleakage than the other groups. In the cervical wall, teeth restored with a flowable composite liner showed less marginal microleakage when compared to all other groups.

**Conclusion:**

Based on the results of this study, the use of flowable composite as the first increment is recommended in deep class II cavities.

## INTRODUCTION

Amalgam was the material of choice worldwide for class I and class II restorations for more than a century. Declining acceptance of amalgam and patients’ interest in dental esthetics resulted in the development of new tooth colored restoratives and techniques.[1]

Composites were introduced in the 1960's and since then have undergone a lot of research and development. Refinement in these materials led to the development of microhybrid composite, with mean particle sizes in the 0.6- to 0.7-micrometer range.[2] Although successful techniques for posterior resin placement have been developed with these universal microhybrid materials, they present many challenges when clinicians attempt to place morphologically correct and functional class II restorations.[3] Many hybrid composites are difficult to manipulate because of inherent stickiness and slumping.[3]

“Packable” composites are introduced to marketplace as an alternative to amalgam. Packables have higher filler loadings (> 80% by weight); therefore, they tend to feel stiffer than traditional composites and handle more like amalgam. Due to their packability, these composites help in restoring good contacts in posterior teeth.[2,3] These stiffer materials may not adequately adapt to internal areas and cavosurface margins at the cervical joint. Flowable resin composites used as liners in areas of difficult access have been suggested to address this concern. Introduced in the late 1996, flowable composites were created by retaining the same particle sizes of traditional hybrid composites, but reducing the filler content and allowing the increased resin to reduce the viscosity of the mixture.[4]

Despite advances that have been made, many clinical and material limitations have restricted the universal use of resin composites as posterior restorative materials. One of the most undesirable characteristics of composite resin is its polymerization shrinkage.[5] Stress form shrinkage-strain can cause clinical problems such as postoperative pain, fracture of the tooth and opening of the restoration margins that can result in microleakage and recurrent caries.[6] This microleakage can be expected and frequently detected on the proximal gingival margins of class II restorations, in which little or no enamel remains.[7]

Many techniques have been tried to reduce this microleakage. Oblique layering technique with increments (1 to 1.5 mm in depth) of wedge- or triangle-shaped produce lowest C factor and minimizes stress formation.[8] When used as lining materials beneath composite restorations, flowable composites may improve marginal adaptation. A recent method designed to reduce shrinkage stress consists of reducing the initial conversion by using different curing modes. An exponential increase of intensity over a given period in ramped or soft start curing technique provides the least shrinkage stress and potentially the optimized polymerized state.[9] The use of beta-quartz glass ceramic inserts[10] and pre-polymerized composite inserts[11] in the mass of restorative material are other methods proposed to minimize polymerization shrinkage of light-cure composites.

However, there is lack of abundant literature on comparative use of flowable liner or a pre-cured composite insert in decreasing the microleakage of composite resin restorations. So, this study is designed to compare the effect of flowable liner and precured composite insert in reducing marginal microleakage of two different composites (Microhybrid and Packable) in Class II cavities, where oblique layering technique and ramp curing mode are used during restoration.

## MATERIALS AND METHODS

**Figure 1:**
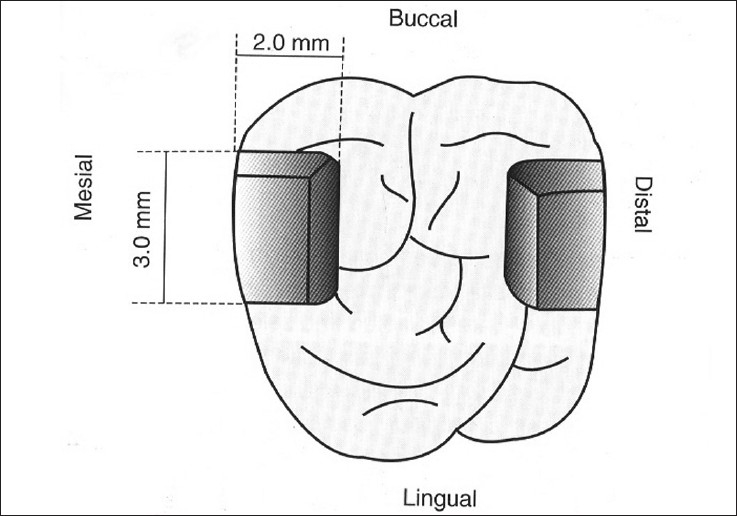
Schematic representation of the cavity preparation dimensions, at occlusal view

**Figure 2:**
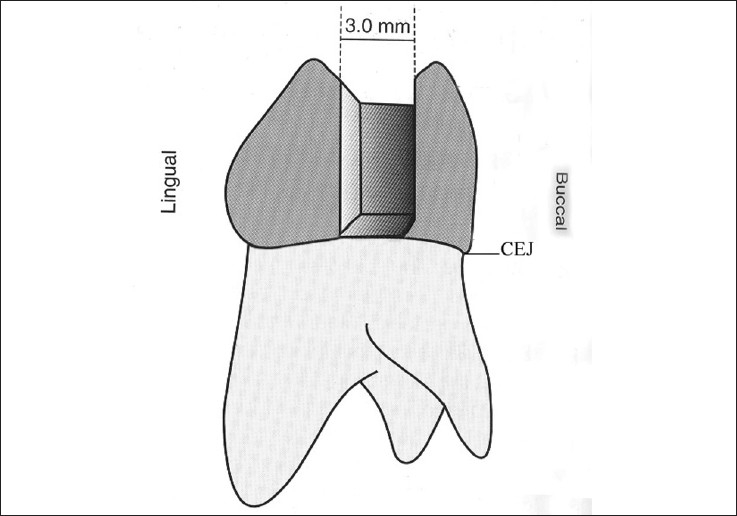
Schematic representation of the cavity preparation dimensions, at proximal view

Sixty human third molars free of caries and restorations were collected and stored in normal saline. One hundred and twenty standardized class II cavities were prepared on the mesial and distal surfaces of each tooth. Each cavity was prepared with a carbide bur (# 245, SS White). For every five preparations, a new bur was used. The final preparation showed the following dimensions: 2.0 mm occlusal extension, 3.0 mm buccal-lingual extension. The gingival seat was placed at the CEJ [Figure 1 and 2]. Each specimen was mounted with the adjacent teeth for placement of Tofflemire matrix which allowed building up of the proximal wall.

All cavity surfaces were dried with oil free compressed air followed by etching with 35% phosphoric acid for 15 seconds, rinsed with water for 15 seconds and excess water was removed with the help of a tissue paper, leaving a glistening hydrated surface. The primer (Scotch Bond Multipurpose, 3M Espe) was applied to etched enamel and dentin and gently dried for 5 seconds. Then, the Scotch Bond Multipurpose adhesive was applied to primed enamel and dentin and light cured for 10 seconds. The specimens were randomly divided into six groups.

In all the groups, each increment was cured for 40 seconds using the ramp curing mode (initial intensity of 200 mW/cm^2^ and increasing over 600 mW/cm^2^ in 40 seconds). The thickness of the first increment was 0.5 mm and this increment was cured for an additional 20 seconds using conventional curing mode. The thickness of each increment placed was confirmed with the help of a graduated probe by measuring the depth of the walls prior to and after the placement of the composite. The rest of the cavity was filled with the help of oblique layering technique, i.e. using triangular or wedge shaped increments of 1.5 mm thickness, which contacted only one opposing wall at a time. The second and third increments were cured for an additional 10 seconds. The curing was done from the occlusal aspect with the tip of the curing unit placed as close to the occlusal surface as possible.

Group (G1) was restored with the microhybrid resin composite Z 100 (3M, Espe) using oblique layering technique. Group 2 (G2) was restored with the Packable/compactable resin composite Filtek P60 (3M Espe), using oblique layering technique. Group 3 (G3) was restored with a first increment of a flowable resin composite, Filtek Flow (3M Espe), placed on the cervical wall of the cavity in a thickness of 0.5 mm and light cured for an additional 20 seconds using the conventional curing mode. The rest of the cavity was then restored with microhybrid resin composite (Z100, 3M) using the oblique layering technique. Group 4 (G4) was restored with the first increment of a flowable resin as described in group 3. The rest of the cavity was restored with packable resin composite (Filtek P60) using the oblique layering technique. Group 5 (G5) and Group 6 (G6) were restored in the similar way as groups 1 and 2 but with a precured composite insert in the second increment. The pre-cured composite insert was prepared using a Teflon mold of 1mm diameter and 1mm depth. Resin composite was placed into the mold using a smooth surface condenser. The excess composite was removed using a scalpel blade and the surfaces of the resin composite inserts were immediately covered with a transparent cellulose strip and cured for 40 seconds. Then the insert was removed from the mold.

Following the restorative procedures, the metallic individual matrix was removed and the occlusal surface finished and polished. The teeth were now mounted in a 0.75 inch diameter polyvinyl chloride (PVC) ring filled with auto-polymerized acrylic resin, to allow root sealing. The specimens were then kept in distilled water at 370° C for 24 hours. In order to evaluate the marginal microleakage, the teeth surfaces were isolated with nail varnish, except for 2.0 mm around the restoration to allow contact with the tracing agent. The specimens were thermocycled at 5 ± 10° C and 55 ± 10° C for 1,000 cycles and immersed in 50% silver nitrate solution for 2 hours. Following removal of nail varnish, the specimens were sectioned using microtome at the center of the restoration mesiodistally. Each of the sectioned surfaces was regularized with Super Snap polishing discs. The sectioned specimens were observed with a steromicroscope (X30 magnification) and scored for the degree of dye penetration at the occlusal and cervical walls.

### Scoring for dye penetration for marginal microleakage on the occlusal wall:

0 – No dye penetration

1 – Dye penetration into enamel

2 – Dye penetration beyond the dentinoenamel junction

3 – Dye penetration into the pulpal wall

### Scoring for dye penetration for marginal microleakage on the cervical wall:

0 – No dye penetration

1 – Dye penetration into half extension of cervical wall

2 – Dye penetration into more than half or complete extension of the cervical wall

3 – Dye penetration into cervical and axial walls toward the pulp

**Figure 3:**
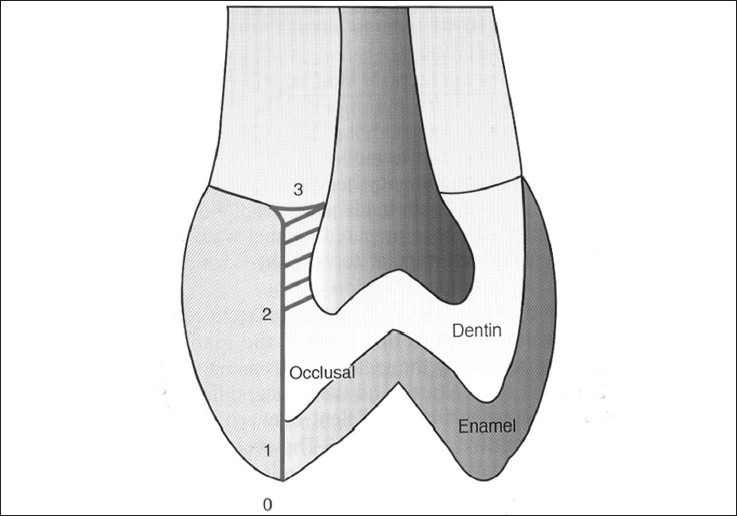
Degrees of dye penetration for marginal microleakage on the occlusal wall

**Figure 4:**
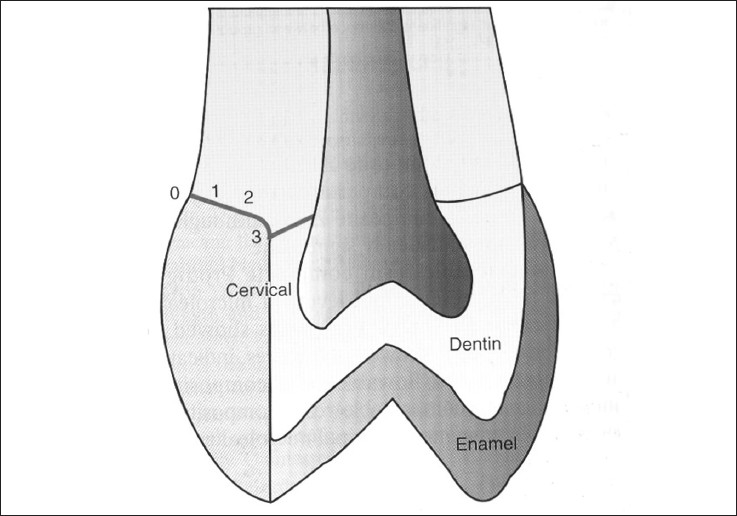
Degrees of dye penetration for marginal microleakage on the cervical wall

These are illustrated in Figures 3 and 4.

## RESULTS

The aim of the present study was to evaluate marginal microleakage in class II composite restorations at the occlusal and cervical margins. Multiple group comparison was done using ANOVA. Mann Whitney test and Chi-square test were used to calculate *P* value among different test groups. If *P* < 0.05, it indicates significant difference. If *P* < 0.001, it indicates highly significant difference among different groups.

In the occlusal margin, except for groups 2 and 6, the restorations in the other groups were relatively free of marginal microleakage. Groups 3 and 4 showed no microleakage in the occlusal wall. Group1 showed dye penetration into enamel in one cavity and beyond dentino-enamel junction in one cavity. Group 5 showed dye penetration into enamel in 2 cavities. The microleakage on the occlusal wall is not significant statistically between Groups 1, 3, 4 and 5 when analyzed with ANOVA.

The order of microleakage can be expressed as follows: 3 = 4 < 5 < 1 < 6 < 2

At the cervical margin, it was seen that restorations free of microleakage were hard to achieve. The microleakage in the cervical margin was seen in all the groups. The order of microleakage in the cervical wall is as follows: 3 < 4 < 5 < 6 < 1 < 2

**Graph 1:**
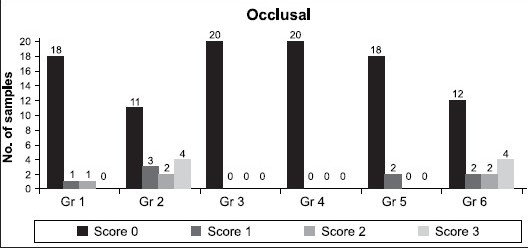
Number of teeth showing different microleakage scores in the occlusal wall

Table 1 shows the dye penetration score as an indicator of microleakage in the occlusal wall. Results for occlusal wall showed significantly better score for groups 3 and 4, when compared to other groups. The order of microleakage is as follows: 3 = 4 < 5 < 1 < 6 < 2. The same results were depicted in Graph 1.

**Table 1:** Frequency of dye penetration score as an indicator of marginal microleakage in occlusal wall

Groups	N	Score				MEAN ± SD
		0	1	2	3	
1	20	18	1	1	-	0.15 ± 0.49
2	20	11	3	2	4	0.95 ± 1.23
3	20	20	0	0	0	0.0
4	20	20	0	0	0	0.0
5	20	18	2	0	0	0.10 ± 0.31
6	20	12	2	2	4	0.90 ± 1.25

Mann Whitney and Chi-Square test, *P* < 0.05 significant difference, *P* < 0.001 highly significant difference, *P* > 0.05 not significant difference, Significant difference between groups: 1 – 2, 1 – 6, 2 – 3, 2 – 4, 2 – 5, 4 – 6, 5 – 6

**Graph 2:**
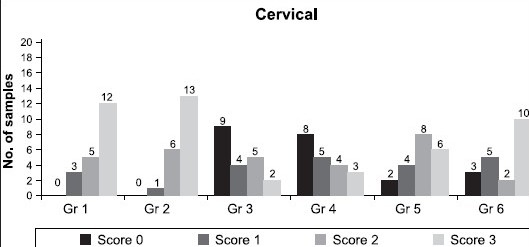
Number of teeth showing different microleakage scores in the cervical wall

Table 2 shows the dye penetration score as an indicator of microleakage in the cervical wall. Results for cervical wall showed highly significant difference in the groups 3 and 4, when compared to groups 1 and 2. Significant difference was seen in groups 5 and 6, when compared to 1 and 2. The order of microleakage in the cervical wall is as follows 3 < 4 < 5 < 6 < 1 < 2. The same results are depicted in Graph 2.

**Table 2:** Frequency of dye penetration score as an indicator of marginal microleakage in cervical wall

Groups	N	Score				MEAN ± SD
		0	1	2	3	
1	20	0	3	5	12	2.45 ± 0.76
2	20	0	1	6	13	2.60 ± 0.60
3	20	9	4	5	2	1.00 ± 1.08
4	20	8	5	4	3	1.10 ± 1.12
5	20	2	4	8	6	1.90 ± 0.97
6	20	3	5	2	10	1.95 ± 1.19

Mann Whitney test, *P* < 0.05 significant difference, *P* < 0.001 highly significant difference, *P* > 0.05 not significant difference, Significant difference between groups: 1 – 3, 1 – 4, 1 – 5, 2 – 3, 2 – 4, 2 – 5, 3 – 5, 3 – 6, 4 – 5, 4 – 6

## DISCUSSION

In recent years, resin composites have become more useful as posterior restorative materials. Polymerization shrinkage is one of the primary deficiencies that complicated the use of this versatile class of restorative material and possibly limits service life expectations.

Polymerization shrinkage of dental resin composites is because monomer molecules are converted into a polymer network and, therefore, exchange van der Waals spaces into covalent bond spaces. This polymerization shrinkage creates contraction stresses in the resin composite restoration leading to microleakage and internal stress in the surrounding tooth structure.[5,6] Reduction of the polymerization shrinkage has been an important issue with the use of dental resin composites.[12] Thus, in spite of much advancement seen in the composite restoratives and bonding agents, reliable adhesion without marginal gap formation has proven elusive.

Microleakage is defined as the clinically undetectable passage of bacteria, fluids, molecules, or ions between a cavity wall and the restorative material applied to it.[13] Microleakage at the tooth-restoration interface is considered a major factor influencing the longevity of dental restorations. It may lead to staining at the margins of the restoration, a hastening of the breakdown at the margins/restorations, recurrent caries at the tooth/restoration interface, hypersensitivity of the restored teeth, and the development of pulpal pathology.[14] Furthermore, the differences in the coefficients of thermal expansion of the tooth and the resin composite can lead to different volumetric changes in the resin and the tooth structure during temperature changes.[15] The different volumetric changes directly affect microleakage.[16]

Restoring class II cavities with resin composites has always been a point of debate, especially in deep cavities where there is no enamel and the cavity margins are formed of dentin, cementum or both. Bonding to dentin is more difficult to achieve due to the specific properties of dentin such as tubular structure and intrinsic wetness.[17] To minimize stress from polymerization shrinkage, efforts have been directed towards improving placement techniques, composite formulations, and curing methods.[18] So, the present study is designed to compare the effect of flowable composite liner and precured composite insert in reducing marginal microleakage of two different composites (Microhybrid and Packable) in class II cavities, where oblique layering technique and ramp curing mode were used.

The use of oblique layering technique limits the development of contraction forces between opposing walls, which could be detrimental to the restoration quality (stress build-up, gap formation, and cuspal fissures).[8] Hence, in this study the oblique layering technique was used. To compensate for the loss of curing light intensity in deep class II cavities, the layers of composite that are at a greater distance from the light rod were cured for longer periods of time.[19]

In this study, in the occlusal wall, Group 2 and 6 (packable composite) showed significant microleakage when compared to other groups. The other four groups did not show any significant difference in the results. The microleakage in groups 2 and 6 occurs due to the increase in the amount of filler particles and a consequent reduction in viscosity of the resin composite, leading to an inadequate adaptation to the enamel walls.[20] Also, the packable composites have insufficient matrix available for wetting the cavity wall and melting of the subsequent layers leading to formation of voids.[21] Voids in the restoration set the stage for postoperative sensitivity and bacterial microleakage. These voids may cause the restoration to fail and lead to caries and possible pulp involvement.[22]

The reliable enamel bond with the restoration in group 1 (Microhybrid composite)[23] and the presence of precured composite insert which decreased the amount of uncured composite within the cavity (around the inserts)[24] in group 5 (Microhybrid composite + precured composite insert) reduced the overall polymerization shrinkage and the stress associated in groups 1 and 5. This good seal with enamel margins is because of the enamel etching technique in controlling microleakage.[10] Also the thin and medium consistency composites (Flowable composite liner in groups 3 and 4) have better adaptability when compared to packable composites whatever the application mode used.[25] In the cervical wall, the order of microleakage is as follows: 3 < 4 < 5 < 6 < 1 < 2. Group 1 and group 2 showed the maximum microleakage in most of the cavities. This was due to the unreliable bond produced by both the restorative materials (Microhybrid and Packable) with the dentin and/or cementum. Additionally the shrinkage of resin during polymerization can overcome the bond to dentin and produce micro gaps.[26] When these two groups are compared, group 1 showed less microleakage than group 2. This is because Microhybrid composite has better adaptability than Packable composite due its less viscosity and hence better flow.

Groups with flowable composite liner, (groups 3 and 4), showed the least amount of leakage in the cervical wall though they could not completely prevent leakage. The flowable composite used as a liner underneath the composite restoration has several advantages. Firstly, the flowable composites are dispensed from syringe and can flow into the preparation, resulting in greater ease of placement and allowing the dentist to cover the entire preparation. This more accurate method of insertion reduces the possibility of voids at the interface.[27]

Secondly, the flowable composite liner may act as a flexible intermediate layer, which helps relieve stresses during polymerization shrinkage of the restorative resin.[28,29] This is due to the low Young's Modulus of the flowable composites in comparison to the other hybrids.[30,22] This could contribute to the dissipation of contraction stresses during polymerization.[30]

One of the most influential factors for microleakage is the alternating contraction and expansion of the restorative material when subjected to changes in temperature.[15] Since it has less filler content, the coefficient of thermal expansion of flowable composite is close to that of the tooth structure[30,31] and this further increased the marginal adaptation when the specimens were thermocycled.

In the cervical wall, the next best results were shown in groups using a precured composite insert (Groups 5 and 6). This precured composite insert reduced the amount of uncured composite within the cavity, therefore reducing the overall polymerization shrinkage and the stress associated with it.[24] In addition, the placement of the pre-cured composite insert within a cavity already partially filled with composite might have improved the adaptation of the composite to the cavity walls.[24]

These results suggest that low viscosity of flowable composite, used as a liner, leads to better adaptation along cavity walls and moreover flowable composite with low modulus of elasticity acts as stress breaker; hence reducing the effect of polymerization shrinkage.[31]

## CONCLUSION

Within the limitations of this study, it can be concluded that the use of a flowable composite liner with microhybrid or packable composites is highly recommended in restoring deep class II cavities to reduce the marginal microleakage and the problems associated with it. Precured composite insert, to some extent also prevents the microleakage.
